# CdS quantum dot-sensitized solar cells based on nano-branched TiO_2_ arrays

**DOI:** 10.1186/1556-276X-9-107

**Published:** 2014-03-04

**Authors:** Chang Liu, Yitan Li, Lin Wei, Cuncun Wu, Yanxue Chen, Liangmo Mei, Jun Jiao

**Affiliations:** 1 School of Physics and State Key Laboratory of Crystal Materials, Shandong University, Jinan 250100, People's Republic of China; 2 School of Information Science and Engineering, Shandong University, Jinan 250100, People's Republic of China; 3 Department of Mechanical and Materials Engineering, Portland State University, P.O. Box 751, Portland, OR 97207-0751, USA

**Keywords:** TiO_2_, CdS, Nanobranch, Solar cells

## Abstract

Nano-branched rutile TiO_2_ nanorod arrays were grown on F:SnO_2_ conductive glass (FTO) by a facile, two-step wet chemical synthesis process at low temperature. The length of the nanobranches was tailored by controlling the growth time, after which CdS quantum dots were deposited on the nano-branched TiO_2_ arrays using the successive ionic layer adsorption and reaction method to make a photoanode for quantum dot-sensitized solar cells (QDSCs). The photovoltaic properties of the CdS-sensitized nano-branched TiO_2_ solar cells were studied systematically. A short-circuit current intensity of approximately 7 mA/cm^2^ and a light-to-electricity conversion efficiency of 0.95% were recorded for cells based on optimized nano-branched TiO_2_ arrays, indicating an increase of 138% compared to those based on unbranched TiO_2_ nanorod arrays. The improved performance is attributed to a markedly enlarged surface area provided by the nanobranches and better electron conductivity in the one-dimensional, well-aligned TiO_2_ nanorod trunks.

## Background

Solar cells have attracted considerable attention because of their potential application in low-cost and flexible energy generation devices. Since the seminal work pioneered by O'Regan and Grätzel in 1991, dye-sensitized solar cells have been investigated extensively all over the world [1-11]. Assembly of branched nanostructures also received intense scrutiny due to their potential effects to a number of promising applications such as solar cells, water splitting, optoelectronics, sensing, field emission, and more [12,13]. In 2013, Roh et al. studied solar cells based on nano-branched TiO_2_ nanotubes, specifically, nanotubes characterized by increased surface area [14]. The results were attractive; they were able to achieve an impressive light-to-electricity conversion rate. Also of note, Roh et al. used organic dye as a sensitizer to fabricate solar devices. However, the use of dye as a sensitizer is problematic for two reasons: first, organic dye is expensive; second, and perhaps more importantly, the organic dye proved to be unstable. As a result, using dye to sensitize solar cells is still not feasible for practical applications.

Because it is critical to tailor materials to be not only cost-effective but also long-lasting, inorganic semiconductors such as CdSe [15,16], PbS [17-19], CdS [20], and Sb_2_S_3_[21,22] have several advantages over conventional dyes: first, the band gap of semiconductor nanoparticles can be tuned by size to match the solar spectrum; second, their large intrinsic dipole moments can lead to rapid charge separation and a large extinction coefficient, which is known to reduce the dark current and increase the overall efficiency; third, and finally, semiconductor sensitizers provide new chances to utilize hot electrons to generate multiple charge carriers with a single photon. Hence, nano-sized, narrow band gap semiconductors are ideal candidates for the optimization of solar cells to achieve improved performance. To date, CdS-sensitized solar cells have been studied by many groups [23-26]. In most reported works, CdS quantum dots were grown on TiO_2_ nanotubes and TiO_2_ nanoporous photoanodes with hierarchical pore distribution. However, little work has been carried out on utilizing nano-branched TiO_2_ arrays as photoanodes. Compared to polycrystal TiO_2_ nanostructures, such as nanotubes and nanoparticles, nano-branched TiO_2_ nanorod arrays, which are grown directly on transparent conductive oxide electrodes, increase the photocurrent efficiency by avoiding the particle-to-particle hopping that occurs in polycrystalline films. These nanostructures could simultaneously offer a large surface area for deposition of CdS quantum dots, excellent light-trapping characteristics, lower charge carrier recombination rates, and a highly conductive pathway for charge carrier collection, resulting in a highly efficient photoanode for solar cell applications.

In this study, a facile, two-step wet chemical synthesis process at low temperature was applied to vertically grown TiO_2_ nano-branched arrays on F:SnO_2_ conductive glass (FTO). By varying the growth time, the length of nanobranches was optimized to provide a larger area for deposition of CdS quantum dots. Using the successive ionic layer adsorption and reaction (SILAR) method, CdS quantum dots were deposited on the surface of TiO_2_ nano-branched arrays to make a photoanode for quantum dot solar cells. The efficiency of the solar cells varied as the growth time of TiO_2_ nanobranches changed. A light-to-electricity conversion efficiency of 0.95% was recorded for solar cells based on an optimized nano-branched array, indicating an increase of 138% compared to that of solar cells based on unbranched arrays.

## Methods

### Growth of single-crystalline rutile TiO_2_ nano-branched arrays by facile, two-step wet chemical synthesis process

The TiO_2_ nanorod arrays were obtained using the following hydrothermal methods: 50 mL of deionized water was mixed with 40 mL of concentrated hydrochloric acid. After stirring at ambient temperature for 5 min, 400 μL of titanium tetrachloride was added to the mixture. The feedstock prepared above was injected into a stainless steel autoclave with a Teflon lining. The FTO substrates were ultrasonically cleaned for 10 min in a mixed solution of deionized water, acetone, and 2-propanol with volume ratios of 1:1:1 and were placed at an angle against the Teflon liner wall with the conducting side facing down. The hydrothermal synthesis was performed by placing the autoclave in an oven and keeping it at 180°C for 2 h. After synthesis, the autoclave was cooled to room temperature under flowing water, and the FTO substrates were taken out, washed extensively with deionized water, and dried in the open air.

The TiO_2_ nanobranches were grown by immersing the TiO_2_ nanorod arrays prepared above in a bottle filled with an aqueous solution of 0.2 M TiCl_4_. The bottle was sealed and kept at a constant temperature of 25°C for 6 to 24 h. Finally, the TiO_2_ nano-branched arrays on FTO were rinsed with ethanol and air-dried at 50°C. After synthesis, the nano-branched arrays were annealed under 450°C for 30 min.

### Deposition of CdS quantum dots using successive ionic layer adsorption and reaction method

In a typical SILAR deposition cycle, Cd^2+^ ions were deposited from a 0.05 M Cd(NO_3_)_2_ ethanol solution; the sulfide source was 0.05 M Na_2_S in methanol/water (1:1, *v*/*v*). The conductive FTO glass, pre-grown with TiO_2_ nano-branched arrays, was dipped into the Cd(NO_3_)_2_ ethanol solution for 2 min, then dipped into a Na_2_S solution for another 5 min. This entire SILAR process was repeated to obtain the optimal thickness of CdS quantum dots.

### Characterization

A field emission scanning electron microscope (FESEM; Hitachi S-4800, Hitachi, Ltd., Chiyoda, Tokyo, Japan) was used to characterize the morphology of the samples. The crystal structure of the TiO_2_ nano-branched arrays was examined by X-ray diffraction (XRD; XD-3, PG Instruments Ltd., Beijing, China) with Cu Kα radiation (*λ* = 0.154 nm) at a scan rate of 4° per min. X-ray tube voltage and current were set to 36 kV and 20 mA, respectively. The optical absorption spectrum was obtained using a UV-visible spectrometer (TU-1900, PG Instruments, Ltd., Beijing, China).

### Solar cell assembly and performance measurement

Solar cells were assembled using nano-branched TiO_2_/CdS nanostructures as photoanodes. Pt counter electrodes were prepared by depositing a 20-nm-thick Pt film on FTO glass using magnetron sputtering. A 60-μm-thick sealing material (SX-1170-60, Solaronix SA, Aubonne, Switzerland) with a 5 × 5 mm^2^ aperture was pasted onto the Pt counter electrodes. The Pt counter electrode and the nano-branched TiO_2_/CdS photoelectrode were sandwiched and sealed with the conductive sides facing inward. A polysulfide electrolyte was injected into the space between the two electrodes. The polysulfide electrolyte was composed of 0.5 M sulfur, 1 M Na_2_S, and 0.1 M NaOH, all of which were dissolved in methanol/water (7:3, *v*/*v*) and stirred at 80°C for 2 h.

A solar simulator (Model 94022A, Newport, OH, USA) with an AM1.5 filter was used to illuminate the working solar cell at a light intensity of 1 sun illumination (100 mW/cm^2^). A sourcemeter (2400, Keithley Instruments Inc., Cleveland, OH, USA) provided electrical characterization during the measurements. Measurements were calibrated using an OSI standard silicon solar photodiode.

## Results and discussion

**Figure 1:**
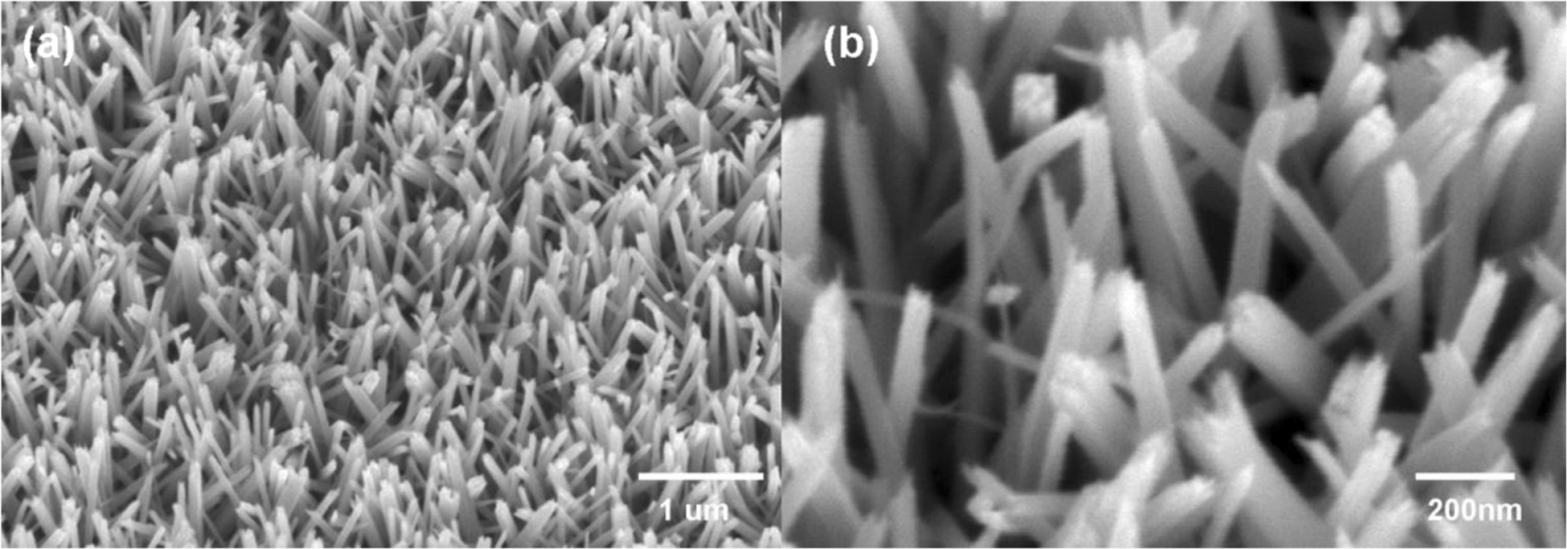
**Typical FESEM images of bare TiO**_**2 **_**nanorod arrays at (a) low and (b) high magnifications.**

**Figure 2:**
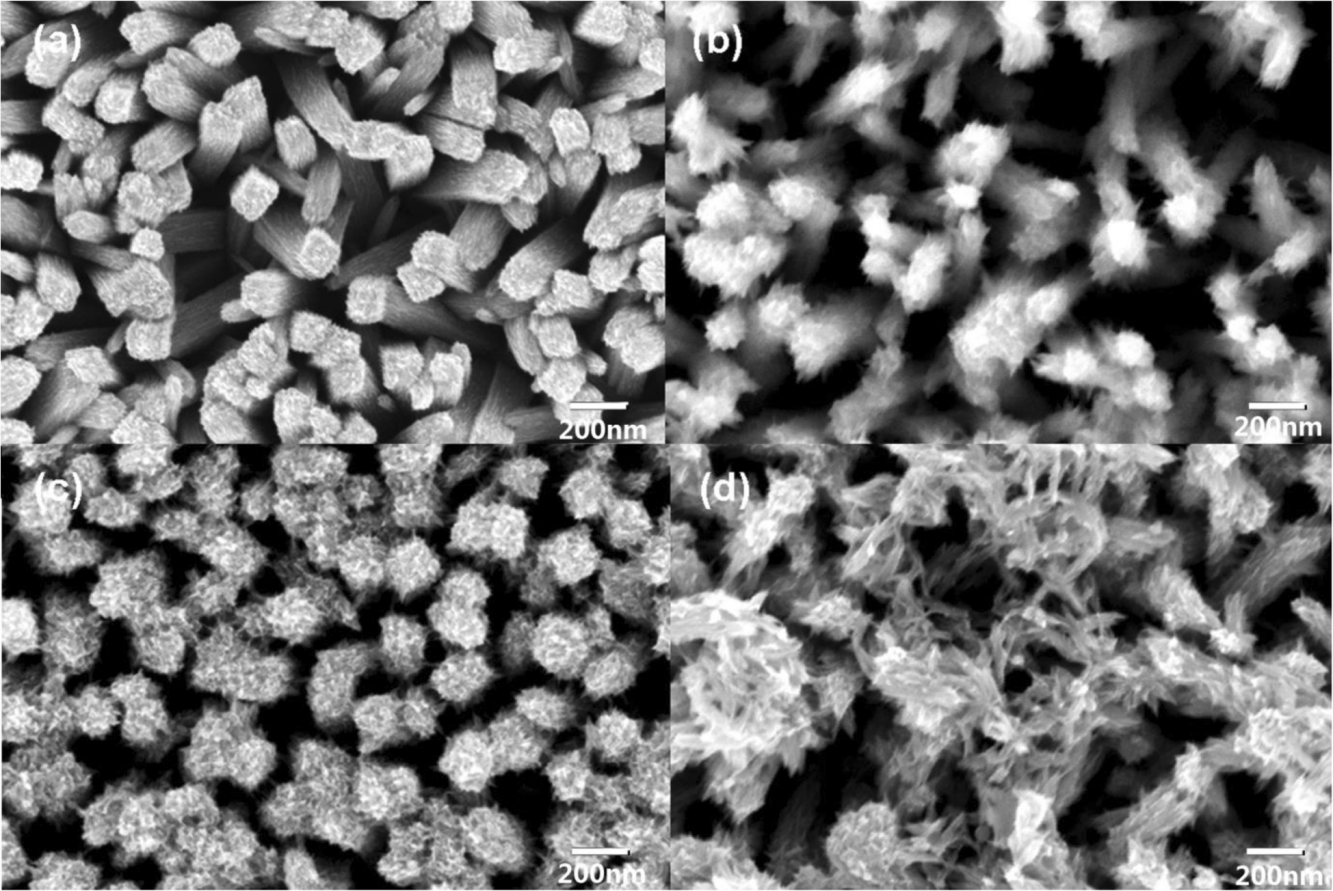
**Morphologies of TiO**_**2 **_**nano-branched arrays.** FESEM images of TiO_2_ nano-branched arrays synthesized via immersing TiO_2_ nanorod arrays into an aqueous TiCl_4_ solution for **(a)** 6, **(b)** 12, **(c)** 18, and **(d)** 24 h.

Figure 1 shows the typical FESEM images of TiO_2_ nanorod arrays on FTO-coated glass substrates, at both (a) low magnification and (b) high magnification. It can be observed that the FTO-coated glass substrate was uniformly covered with ordered TiO_2_ nanorods. The density of the nanorods was 20 nanorods/μm^2^, which allows suitable space for growth of TiO_2_ nanobranches. After immersion in an aqueous TiCl_4_ solution for a period of time ranging from 6 to 24 h, nanobranches appeared along the trunks of the TiO_2_ nanorods. The morphology of the branches, shown in Figure 2, is strongly dependent on the amount of time the nanorods remain immersed in the TiCl_4_ solution. As the immersion time increases, the branches become greater in number and longer in length. These branches coated on TiO_2_ nanorod would greatly improve the specific surface area and roughness, which is urgent for solar cell applications. However, when immersed for 24 h or more, the branches form continuous networks that greatly suppress the effective surface area, preventing the CdS quantum dots from fully contracting with the TiO_2_ and therefore decreasing the overall photovoltaic performance.

**Figure 3:**
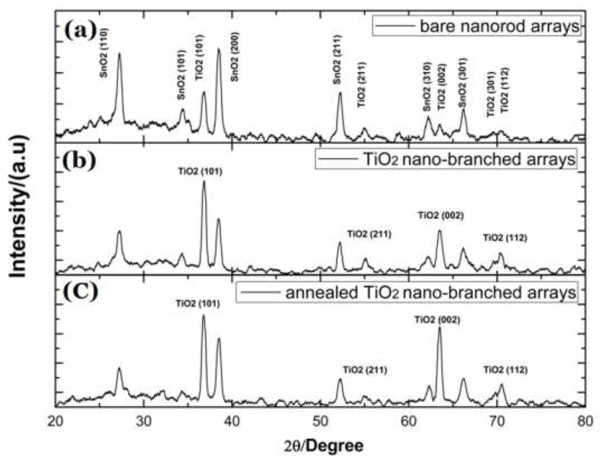
**XRD patterns of TiO**_**2 **_**nanorod and nano-branched arrays.** TiO_2_ nanorod arrays **(a)** and nano-branched arrays without **(b)** and with **(c)** annealing treatment on FTO.

Figure 3 shows XRD patterns of (a) TiO_2_ nanorod arrays and (b) nano-branched arrays without and (c) with annealing treatment, each on FTO. As illustrated in Figure 3a, with the exception of the diffraction peaks from cassiterite-structured SnO_2_, all the other peaks could be indexed as the (101), (211), (002), (310), and (112) planes of tetragonal rutile structure of TiO_2_ (JCPDS no. 02–0494). The formation of rutile TiO_2_ nanorod arrays could be attributed to the small lattice mismatch between FTO and rutile TiO_2_. Both rutile and SnO_2_ have near-identical lattice parameters with *a* = 0.4594 nm, *c* = 0.2958 nm and *a* = 0.4737 nm, *c* = 0.3185 nm for TiO_2_ and SnO_2_, respectively, making the epitaxial growth of rutile TiO_2_ on FTO film possible. On the other hand, anatase and brookite have lattice parameters of *a* = 0.3784 nm, *c* = 0.9514 nm and *a* = 0.5455 nm, *c* = 0.5142 nm, respectively. The production of these phases is unfavorable due to a very high activation energy barrier which cannot be overcome at the low temperatures used in this hydrothermal reaction. No new peaks appear in Figure 3b,c, indicating that the TiO_2_ nano-branched arrays are also in a tetragonal rutile phase.

**Figure 4:**
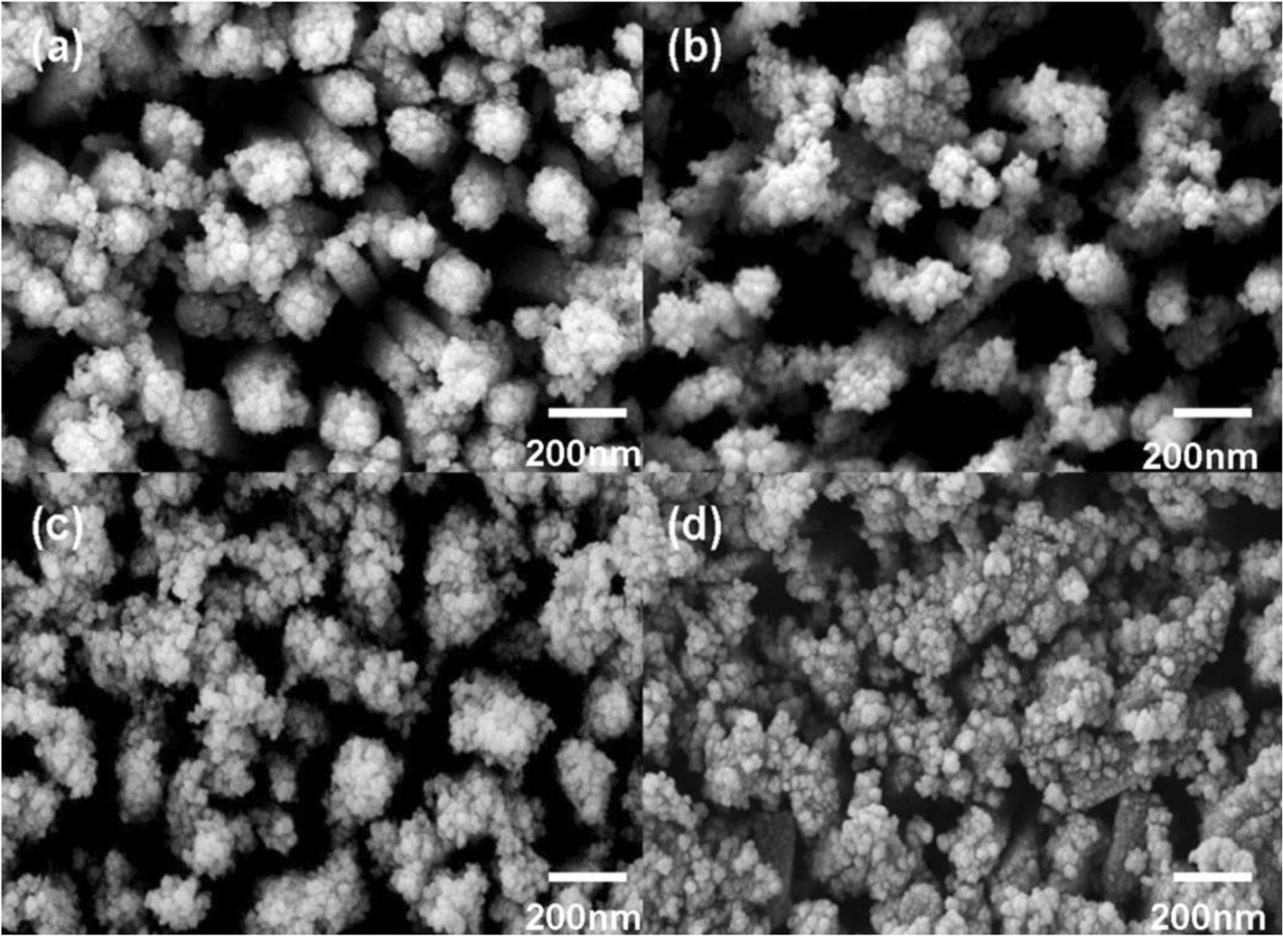
**Morphologies of nano-branched TiO**_**2**_**/CdS nanostructures.** FESEM images of nano-branched TiO_2_/CdS nanostructures with growth time of TiO_2_ nanobranches for **(a)** 6, **(b)** 12, **(c)** 18, and **(d)** 24 h.

CdS quantum dots were deposited on the surface of nano-branched TiO_2_ arrays by SILAR method. The morphologies of CdS/TiO_2_ nano-branched structures were shown in Figure 4. As the length of the nanobranches increased, the space between nano-branched arrays was reduced, indicating that more CdS quantum dots were deposited on the surface of the arrays. For the sample which was immersed in the TiCl_4_ solution for a full 24 h, a porous CdS nanoparticle layer formed on the surface of the TiO_2_ nano-branched arrays. As discussed later, this porous CdS layer causes a dramatic decrease in the photocurrent and efficiency for solar cells.

**Figure 5:**
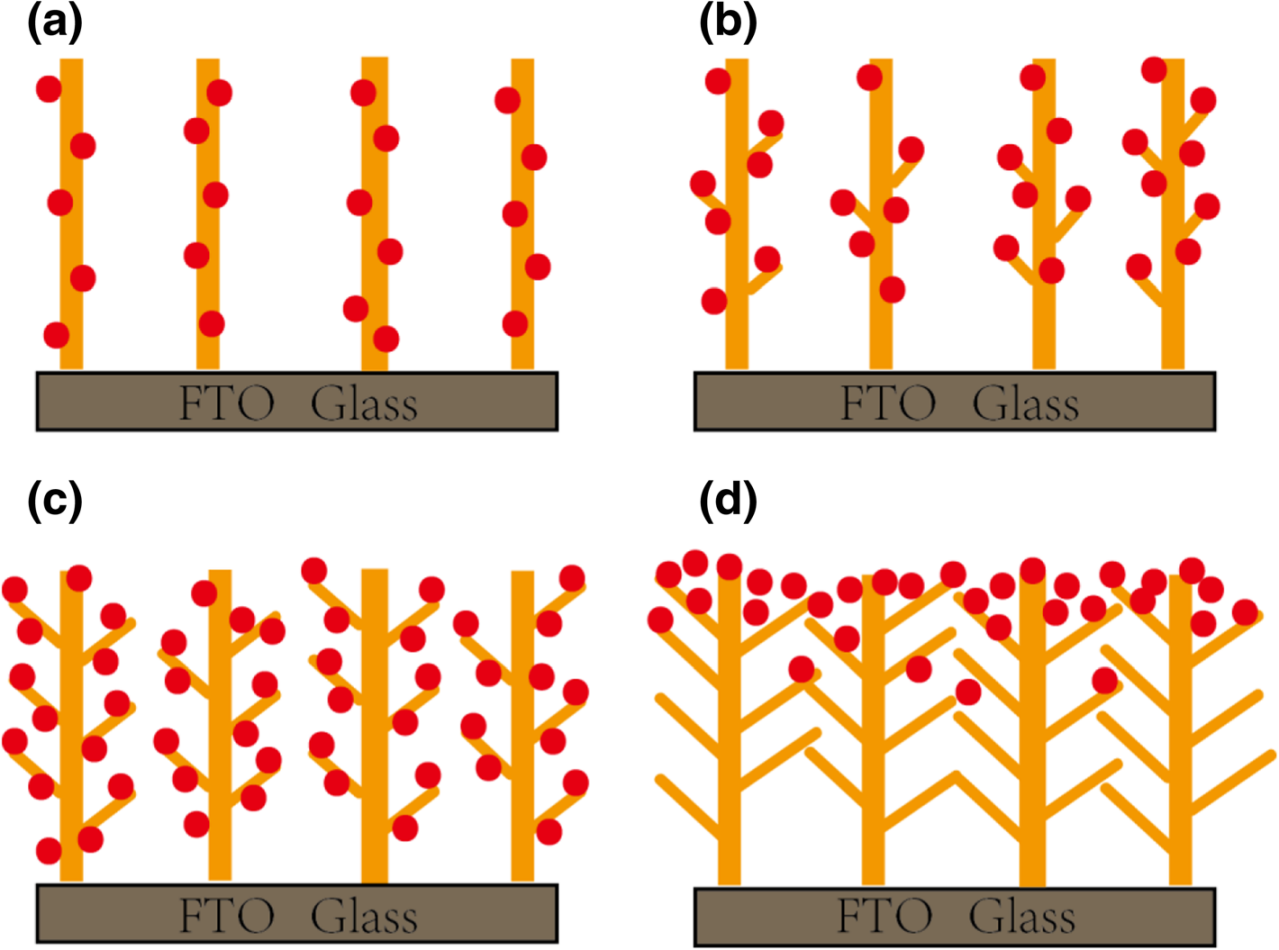
**Schematic of CdS/TiO**_**2 **_**nano-branched structures grown in TiCl**_**4 **_**solution. (a)** 0, **(b)** 12, **(c)** 18, and **(d)** 24 h.

A brief schematic can provide a better impression of these nanostructures. The schematic illustrations of CdS/TiO_2_ nano-branched structures grown in TiCl_4_ solution for (a) 0, (b) 12, (c) 18, and (d) 24 h appear in Figure 5. As the length of nanobranches increased, more contract area was provided for the deposition of CdS quantum dots. However, once the deposition time reached the 24-h mark, the nanobranches intercrossed or interconnected with one another, preventing the CdS quantum dots from making robust connections with the TiO_2_ nano-branched arrays. Once this occurred, a CdS layer then formed a cap on top of the nano-branched TiO_2_ array, resulting in the decrease of the photocurrent and the efficiency of the solar cells.

**Figure 6:**
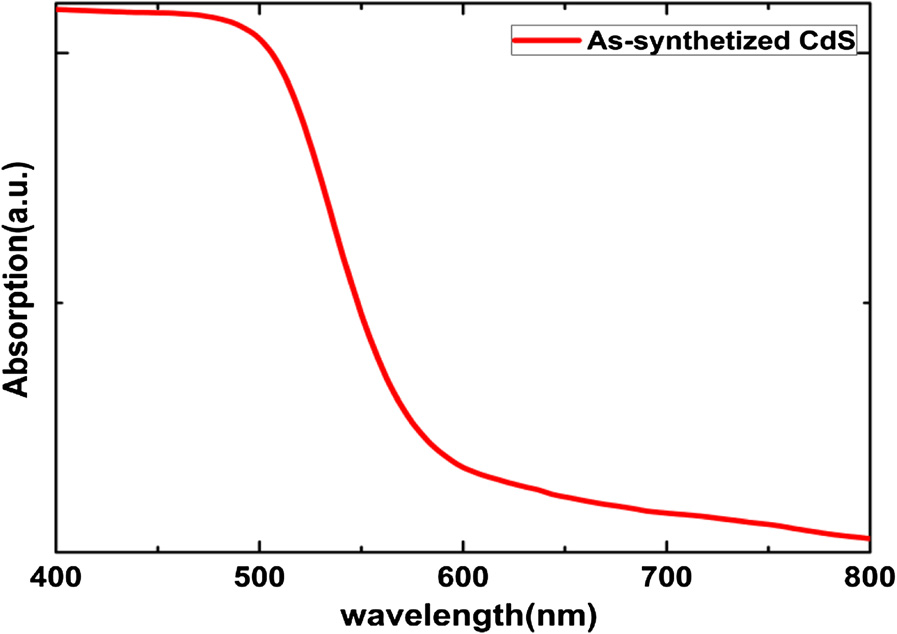
**Typical optical absorption spectra of CdS/TiO**_**2 **_**nano-branched structures.**

The typical UV-visible absorption spectrum of CdS/TiO_2_ nano-branched structure sample is shown in Figure 6. An optical band gap of 2.34 eV is estimated for the as-synthesized CdS quantum dots from the absorption spectra, which closely mirrors the band gap of bulk CdS. No obvious blueshift caused by quantum confinement is observed, indicating the size of the CdS grains is well above the CdS Bohr exciton diameter (approximately 2.9 nm). A strong absorption was observed for light with a wavelength shorter than 540 nm, corresponding to the most intensive part of the solar spectrum.

**Figure 7:**
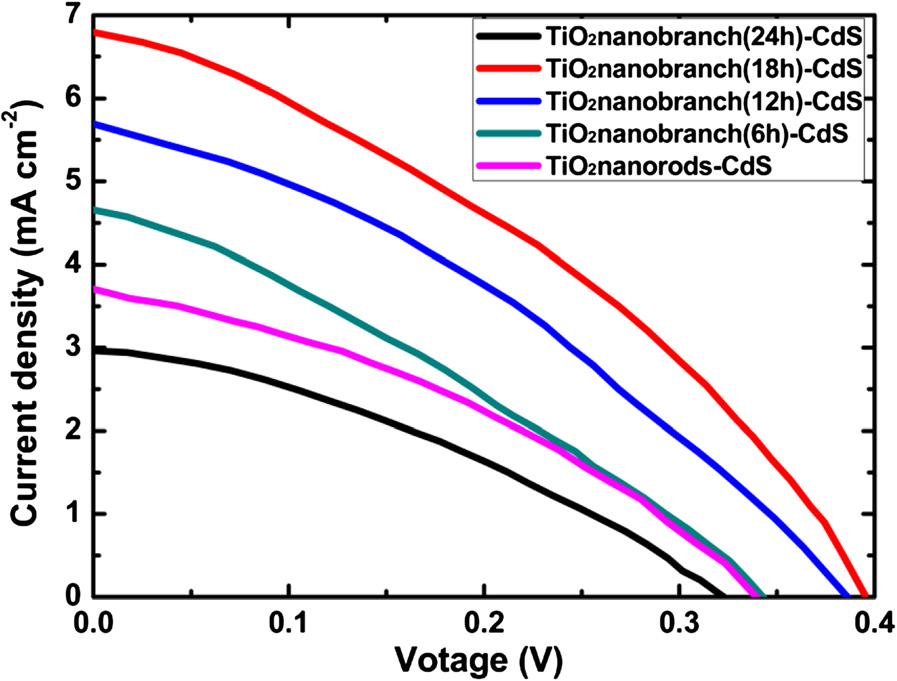
***I*-*V *** **curves for the solar cells assembled using CdS/TiO**_**2 **_**nano-branched structures.**

The photocurrent-voltage (*I*-*V*) performances of the solar cells assembled using CdS/TiO_2_ nano-branched structures grown in TiCl_4_ solution for 6 to 24 h are shown in Figure 7. The *I*-*V* curves of the samples were measured under 1 sun illumination (AM1.5, 100 mW/cm^2^). For solar cells based on bare TiO_2_ nanorod arrays, a short-circuit current density (*J*_sc_) of 3.72 mA/cm^2^, an open voltage of 0.34 V, and an overall energy conversion efficiency of 0.44% were generated. As the growth time of TiO_2_ nanobranches increased from 6 to 18 h, the solar cell performance improved correspondingly. The short-circuit current density (*J*_sc_) improved from 3.72 to 6.78 mA/cm^2^; the open circuit voltage (*V*_oc_) improved from 0.34 to 0.39 V. A power conversion efficiency of 0.95% was obtained for the sample with nano-branched structures grown in TiCl_4_ solution for 18 h, indicating an increase of 138% compared to that based on bare TiO_2_ nanorod arrays. Detailed parameters of the solar cells extracted from the *I*-*V* characteristics are listed in Table 1. As the growth time reaches 24 h or more, the branches on the nanorod arrays were interconnected. The active area of TiO_2_ for CdS deposition decreased, and a porous CdS capping layer formed on top of TiO_2_ arrays. Therefore, excessive long growth time is disadvantageous and leads to a reduced photovoltaic performance of the solar cells.

**Table 1:** ***J***_**sc**_**, *V***_**oc**_**, FF, and efficiency**

	***V***_**oc **_**(V)**	***J***_**sc **_**(mA/cm**^**2**^**)**	**FF (%)**	***η *** **(%)**
TiO_2_ NR/CdS	0.34	3.72	0.35	0.44
TiO_2_ NB (6)/CdS	0.34	4.61	0.32	0.51
TiO_2_ NB (12)/CdS	0.38	5.65	0.37	0.78
TiO_2_ NB (18)/CdS	0.39	6.78	0.36	0.95
TiO_2_ NB (24)/CdS	0.32	3.01	0.34	0.33

*V*_oc_, open-circuit voltage; *J*_sc_, short-circuit photocurrent density; FF, fill factor; *η*, energy conversion efficiency; NR, nanorod arrays; NB, nano-branched arrays.

From the above results, it is clear that solar cells based on the TiO_2_ nano-branched arrays show an improved photovoltaic performance. This significant improvement can be attributed to the following: (1) the specific surface area and roughness factor of TiO_2_ nano-branched arrays were markedly enlarged, leading to expanded areas for the deposition of CdS quantum dots; (2) the photo-generated electrons transport quickly from the TiO_2_ nanobranches through the single-crystalline TiO_2_ nanorods to the FTO substrates, facilitated by the increased electron conductivity of TiO_2_ nanorods; and (3) these nanobranches can fill the gaps between nanorods, which may improve their ability to harvest light, and thereby improve power conversion efficiency.

In our present work, the power conversion efficiency of our solar cells remains too low for use in practical applications. The rather poor fill factor is considered to be the main factor limiting the energy conversion efficiency. This low fill factor may be ascribed to the lower hole recovery rate of the polysulfide electrolyte, which leads to a higher probability for charge recombination. To improve the efficiency of these CdS/TiO_2_ nano-branched quantum dot-sensitized solar cells, a new hole transport medium must be developed, one with suitable redox potential and low electron recombination at the semiconductor-electrolyte interface.

Counter electrodes have also been reported to be another important factor influencing the energy conversion efficiency. Recently, a number of novel materials have been examined and tested as counter electrode materials; these studies prove the influence of various counter electrode materials on the fill factors of solar devices [27-29]. In addition, graphene with outstanding, transparent conducting properties has been explored as an efficient constituent for solar cell applications [30-32]. Further studies will be conducted to optimize the nanostructures and counter electrode materials to improve the performance of our solar cells.

## Conclusion

In this study, large-area nano-branched TiO_2_ nanorod arrays were grown on fluorine-doped tin oxide glass by a low-cost two-step hydrothermal method. The resultant nanostructures consisted of single-crystalline nanorod trunks and a large number of short TiO_2_ nanobranches, which is an effective structure for the deposition of CdS quantum dots. CdS quantum dots were deposited on the nano-branched TiO_2_ nanorod arrays by a successive ionic layer adsorption and reaction method to form an effective photoanode for quantum dot-sensitized solar cells. As the length of nanobranches increased, the conversion efficiency varied respectively. An optimal efficiency of 0.95% was recorded in solar cells based on TiO_2_ nanorod arrays with optimized nanobranches, indicating an increase of 138% compared to those based on bare TiO_2_ nanorod arrays. In this aspect, the nano-branched TiO_2_ arrays on FTO turned out to be more desirable than bare nanorod arrays for the applications of quantum dot-sensitized solar cells. Further studies of both quantum dot-sensitized solar cells and dye-sensitized solar cells based on these hierarchical TiO_2_ nanostructures grown directly on the FTO conductive glass would be promising and significant for solar cell applications.

## Competing interests

The authors declare that they have no competing interests.

## Authors’ contributions

The work presented here was performed in collaboration of all authors. CL and YL carried out the deposition of CdS layers and solar cell assembling and drafted the manuscript. LW carried out the XRD and SEM characterization. CW carried out the photovoltaic performance measurements and the preparation of TiO_2_ nanorod arrays. YC supervised the work and finalized the manuscript. JJ and LM proofread the manuscript and polished the English language. All authors read and approved the final manuscript.
